# Caring for individuals with eating disorders–how to improve care while reducing unnecessary spending?

**DOI:** 10.1371/journal.pmen.0000160

**Published:** 2024-10-28

**Authors:** Claire de Oliveira

**Affiliations:** 1 Centre for Addiction and Mental Health, Campbell Family Mental Health Research Institute, Toronto, Ontario, Canada; 2 Centre for Addiction and Mental Health, Institute for Mental Health Policy Research, Toronto, Ontario, Canada; 3 Management and Evaluation, Dalla Lana School of Public Health, Institute for Health Policy, University of Toronto, Toronto, Ontario, Canada; 4 ICES, Toronto, Ontario, Canada; Uppsala Universitet, SWEDEN

Eating disorders are mental health conditions defined by abnormal eating behaviours that negatively affect a person’s physical and/or mental health. Eating disorders typically include anorexia nervosa, bulimia nervosa, binge eating disorder, and other ‘specified feeding or eating disorders`that do not meet the strict diagnostic criteria of the aforementioned conditions. These disorders have a combined lifetime prevalence of about 7% in women and 3% in men [1] and are frequently associated with psychiatric comorbidities, such as mood and anxiety disorders, post-traumatic stress disorder, and substance use disorders [2], often requiring long-term treatment [3]. Eating disorders can also cause short- and long-term medical complications [4], such as cardiovascular and renal problems, gastrointestinal disturbances, fluid and electrolyte abnormalities, menstrual and fertility problems (among females), osteoporosis and osteopenia, and dental and dermatological problems [5, 6]. Moreover, anorexia nervosa has the highest mortality rate of any psychiatric disorder [7].

Previous studies have shown that the economic burden of eating disorders is substantial [8–10]. Due to the high costs of care in this population, well-organised efforts directed toward early intervention and active management of these individuals’ physical and mental health are warranted. Furthermore, given the surge in eating disorders-related emergency department visits and medical hospitalizations (i.e., acute care) among young women in Canada throughout the pandemic [11], it is important to understand whether there are ways to improve care among this particular population. Many jurisdictions have implemented strategies, such as high-risk care management, to reduce costs and improve the quality of care among patients with high health care needs. High-risk care management involves the provision of intensive, one-on-one services by a health worker, such as a nurse, to patients with complex needs, such as those with an eating disorder. The idea behind these types of strategies/interventions is that the implementation of high-quality outpatient care may help reduce unnecessary acute care for these patients. For example, research suggests that stepped care models, where primary care clinicians play a greater role in service delivery, may be an option to improve patient outcomes in a cost-effective manner [12]. However, it is unclear whether any costs can be reduced, and if so which, especially among patients who require costly care.

## How to reduce unnecessary health care spending?

One potential way to decrease health care spending, without sacrificing high-quality care, may be to target preventable (i.e., potentially unnecessary) acute care among patients with high health care utilisation. Previous work has estimated preventable care among the general population, disease-specific sub-populations, and patients with high health utilisation and costs, but work of this nature has not been carried out for individuals with eating disorders.

Although the economic burden of eating disorders to health care systems is substantial, there is some scope to decrease acute care spending among this patient population. Among a population-based sample of individuals ever hospitalized for an eating disorder, 15% of all acute care spending (i.e., $1,330,839.94 CAD) was for treatable conditions. Among hospitalisations, the highest proportions of preventable care spending were for inpatient episodes related to short-term diabetes complications and urinary tract infections. Among emergency department visits, the highest proportions of preventable care spending were for ambulatory episodes that involved non-emergent conditions (i.e., conditions that do not require immediate medical care within a 12-hour window), such as hip pain, and emergent but primary care treatable conditions (i.e., conditions that require care within a 12-hour window but could have been treated effectively with appropriate primary care management), such as an adverse effect to a drug. By sub-group, preventable acute care spending was higher for females (14%) and those with a diagnosis of bulimia nervosa (21%) [13].

Overall, these findings suggest that providing better diabetes care, which could support individuals in normalizing eating patterns and improving diabetes control [14], could potentially help reduce diabetes-related hospitalisations. Moreover, it is likely that care coordination between health care providers and improved access to primary care and disease prevention, particularly related to diabetes, may help prevent the occurrence of some emergency department visits. Primary care clinicians could play a greater role in delivering care as part of a stepped care model, where patients first receive self-help, and then be “stepped” to outpatient and subsequently further to inpatient care, if they do not respond to the preceding step [12]. These models have been shown to not only improve patient outcomes but also be cost-effective [12]. Cost-savings resulting from the reduction of unnecessary acute care could provide further economic justification for increased investment in outpatient care for individuals with eating disorders.
